# Intermolecular Organization of a Lyotropic Liquid Crystal and Carbon Dot Composite in Microfluidic Channels: Surface and Dynamic Effects

**DOI:** 10.3390/nano15211682

**Published:** 2025-11-06

**Authors:** Artem Bezrukov, Aliya Galeeva, Aleksandr Krupin, Yuriy Galyametdinov

**Affiliations:** Department of Physical and Colloid Chemistry, Kazan National Research Technological University, Kazan 420015, Russia; galeeva-alija@mail.ru (A.G.); krupin_91@mail.ru (A.K.); yugal2002@mail.ru (Y.G.)

**Keywords:** microfluidics, lab on chip, carbon dots, lyotropic liquid crystals, luminescence, anisotropy, lamellar phase, AI, neural network

## Abstract

Composites of lyotropic liquid crystals with biocompatible luminescent nanoparticles represent multifunctional materials with high potential for application in molecular diagnostics and biomedicine. Their integration with microfluidics is a new and scarcely studied approach that offers unique opportunities for tuning properties of such nanomaterials and simulating the biological environment of their application. This paper analyzes the impact of the governing microfluidic factors, including wall effects and flow dynamics, on the intermolecular structure and optical properties of the mesogenic luminescent nanocomposite of tetraethylene glycol monododecyl ether and carbon dots. The nanoscale and microscale surface structure of microchannel walls was found to be the dominating factor for additional near-wall ordering of the intrinsic lamellar structure of the composite. A combination of controlled shear stress with heating–cooling cycles allowed for a gradual and reversible transformation of multilamellar vesicles into the axial lamellar structure, and provided the composite with anisotropic luminescence capabilities according to the study of the luminescent behavior of carbon dots. The collected experimental datasets, comprising hundreds of texture images, allowed for training the neural network for subsequent accurate recognition of the composite nanoscale organization and dynamic properties in straight and serpentine microchannels. The results will contribute to developing AI-powered microfluidic chips with integrated biocompatible nanocomposite materials for testing drug delivery systems and simulating biological capillary environment in organ-on-chip prototypes.

## 1. Introduction

Microfluidics is an area of science and technology that has attracted sustainable and growing interest from researchers in the last two decades because of its microscale approach to various physicochemical processes [1,2,3,4]. Microfluidic confinement offers unique conditions for accurate tuning of intermolecular interactions and supramolecular organization [5,6]. Microfluidic devices are demanded in a range of applications that are based on the delicate control of organized media: nanotechnology [4,7], biotechnology and biomedicine [2,8,9], chemical analysis and molecular diagnostics [10,11,12], drug delivery [13,14,15], testing and screening [16,17,18], and the simulation of processes in human organs [19,20].

Microfluidic chips offer convenient opportunities for individual or simultaneous applications of various physical factors to control supramolecular organization in microchannels. These factors are flow rate [21,22,23], temperature [24], effects of microchannel walls [25,26], geometry [23,27], magnetic and electric fields [28,29,30], etc. Among them, surface and dynamic effects make a predominant contribution to significant differences in processing self-assembled materials in microchips compared to non-microfluidic macroscale conditions [5,6].

Liquid crystals (LCs) represent organized media that can be fine-tuned in microfluidic confinement [31]. The have been intensively studied in microchannels in the last 10–15 years as materials for photonics [32], velocimetry [33], biosensors and biomarkers [34,35], and drug delivery platforms [36]. Among the organized media, liquid crystals are particularly sensitive to surface and dynamic effects in microchannels [32,37,38].

Current trends in liquid crystal microfluidic research follow a general focus on the biomedical applications of microfluidic devices [34,36,39] and require materials with biocompatibility potential such as lyotropic liquid crystals (LLCs) [40], which are structurally similar to biological systems. On the other hand, a key requirement for materials in bioimaging, drug screening, and organ-on-chip platforms is their luminescent capability [41,42,43]. Biocompatible luminescent nanoparticles such as carbon dots [44] and their combinations with LLCs are, therefore, particularly promising for such applications.

Graphene structures and their related materials, which represent the core of carbon dots and carbon quantum dots, are a subject of intensive and focused attention of researchers. These materials possess a set of unique and advanced properties such as absorption characteristics. In [45], the authors present a tunable terahertz perfect absorber based on a bilayer graphene metamaterial with promising applications in communication and high-sensitivity sensing. In [46], the researchers demonstrate a monolayer graphene terahertz absorber for applications in stealth technologies and smart switches in addition to energy absorption and optics.

Compared to thermotropic liquid crystals, LLC microfluidics is the subject of ongoing and active research. Reports are more limited, and are represented by publications in the last 4–5 years [36,47,48,49]. These works predominantly characterize pure LLC systems, while their composites with luminescent media are not primarily considered.

In this respect, an integrated microfluidic study of orientational and luminescent properties of LLCs with doped carbon dots is intended to provide new insights into the properties of such luminescent nanomaterials in confined and dynamic environments. The results may contribute to our understanding of the impact of surface anchoring on the flow of living cells, vesicles, and drug delivery containers in organ-on-chip devices, which simulate the conditions of the human vascular system.

This paper aims to analyze the impact of microchannel walls and flow dynamics on the behavior of the LLC nanocomposite with blue carbon dots (bCDs). We analyzed the orientation and luminescent properties of these composites in microchannels with various geometries and characterized the anchoring of LLC molecules on its walls. We tracked the structural changes in the composite in respect to shear stress in the microchannels. A machine learning approach was applied to process the datasets of the microfluidic experiments and perform a simplified characterization of LLC orientation and dynamics in curved microchannel geometry.

## 2. Materials and Methods

### 2.1. Materials

The lyotropic liquid crystal phase was produced from tetraethylene glycol monododecyl ether—C_12_H_25_(CH_2_CH_2_O)_4_OH or C_12_EO_4_ (Merck, Darmstadt, Germany), 99.999% purity, which was used as received. The LLC samples were prepared by mixing 45 w.% C_12_EO_4_, 5 w.% decanol, and 50 w.% water with 0.1 g/L of pre-dispersed C-dots. Small additions of decanol were used, according to our previous work [50], to expand the concentration range of the lyotropic mesophase in the LLC systems formed by derivatives of ethylene glycol monododecyl ethers, and we continued the experimental series with comparable results. The mixtures were centrifuged at 3000 rpm (25 °C) for 30 min to perform the complete homogenization of the systems. Before further studies, all of the fresh samples were kept at 25 °C for 14 days.

Carbon dots were synthesized using a hydrothermal method [51]. In total, 432 mg o-phenylenediamine and 840 mg citric acid monohydrate were dissolved in 80 mL deionized water, agitated with magnetic stirrer for 10 min, and then autoclaved in a 100 mL Teflon autoclave at 180 °C for 9 h. The reaction product was cooled down and then centrifuged at 10,000 rpm for 10 min. The resulting solution was filtered with a 0.45 µm syringe filter. The filtered solution was further purified with an MW 1000 dialysis membrane (where MW stands for molecular weight) in water for 24 h; the water was changed every hour. The synthesized carbon dots were dried and further used to prepare aqueous solutions.

Microfluidic devices were fabricated using a polydimethylsiloxane (PDMS) Sylgard^TM^ 184 silicone elastomer (Dow Corning Midland, MI, USA) and used as received. This came as a two-part elastomer kit (the pre-polymer and curing agent). SU-8 3050 photoresist (Microchem Corp., Westborough, MA, USA) was used to produce a mold for the microfluidic chips.

### 2.2. Experimental Methods

The characterization of PDMS surfaces was performed by atomic force microscopy (AFM) using a scanning probe microscope NanoEducator by NT MDT (Moscow, Russia). Microfluidic slabs that were used for the fabrication of microfluidic devices were cut to 10–10 mm rectangles after completion of the chip fabrication steps. These slabs were covered with the layer of the composite by gently shearing it with another PDMS slab, and were left for 3 days for drying and further AFM analysis.

Contact angles were measured using the Kruss DSA20 Easy Drop system (Kruss GmbH, Hamburg, Germany). After capturing, all of the images were processed using the Kruss DSA20 Easy Drop software, version V1-03, to evaluate the contact angles.

The orientation behavior of the LLC media in microfluidic flows were studied by polarized optical microscopy using an Olympus BX51 microscope (Olympus, Tokyo, Japan) equipped with a high-precision Linkam heating system that allowed us to provide uniform temperature conditions for the experiments. Microscopy images were captured at 100× and 500× magnification using a ToupCam E3ISPM08300KPC camera (Touptek, Hangzhou, China).

The photoluminescence emission spectra and luminescence anisotropy data of the carbon dots and composite in bulk conditions and inside microfluidic channels were obtained using a Varian Cary Eclipse spectrofluorimeter (Agilent, Santa Clara, CA, USA) with polarization filters. The chip with 300 µm-wide channels were placed into a holder inside the spectrofluorimeter and positioned accordingly to focus the beam on the microchannel in the middle of the chip. Chips with the largest 300 µm width of microchannels were used for experiments to generate a detectable emission signal.

A comparative analysis of the absolute emission intensity of bCDs was challenging to perform due to the following significantly different conditions: bCDs in water, cuvette; bCDs in composite, cuvette; bCDs in water, microfluidic channel; and bCDs in composite, microfluidic channel. Different settings of the spectrofluorimeter (voltages of the photomultiplier) were applied for these different conditions to adequately track the luminescence response of the studied samples. A limitation of these different experimental conditions is that they did not allow us to perform a comparative analysis of the integrated photoluminescence intensity for the results. For comparison of the peaks, we normalized the spectra.

The luminescent properties of the LLC-bCD composites in microchannels were studied by fluorescence microscopy using an Olympus BX43 fluorescent microscope (Olympus, Tokyo, Japan). Microscopy images were captured at 100× magnification using a ToupCam E3ISPM05000KPA camera (Touptek, Hangzhou, China).

### 2.3. Data Processing and Machine Learning Methods

Processing of the luminescence spectra and polarized microscopy images and evaluation of rotation and tilt angles of the LLC molecules was performed using Matlab 2021b software using pre-developed customized scripts. The color profiles of the composite on the microchannel were analyzed by comparing them with a Michel–Levy chart [52]. All of the respective microscopy images were taken at identical settings of both the microscope and the image-capturing software (ToupView, version 4.11) supplied with the camera.

To implement the machine learning approach for evaluating the flow velocity of the composite in microchannels of varying geometries and correlate its polarizing microscopy images with the orientation behavior, the Python TensorFlow package with the Keras interface (software version 2.20) was used. Local computer characteristics, which were used for training the neural network and processing the results, include an Intel Core i7-12700 processor, 48 GB of DDR5 RAM, and a GeForce RTX 2060 graphics processor. The neural network was trained using a dataset of 1000 POM images. Image fragments of 300 × 300 pixels were used for training. After training, the flow velocity and orientation of the liquid crystals in the microchannel were evaluated using a set of 3–5 new sample images from the experimental data.

### 2.4. Fabricating Microfluidic Devices and Performing Microfluidic Experiments

Microfluidic devices were fabricated using standard photolithography techniques [53]. Straight and serpentine chips with rectangular microchannels of 100 µm height and widths 100, 200, and 300 µm were fabricated. SU-8 photoresist and a transparent photomask with a negative image of a microchip were used to produce a 100 µm thick mold of microfluidic chips on top of a 3-inch silicon wafer. A PDMS pre-polymer was mixed with a curing agent, poured over the mold, and cured in an oven for 4 h in 60 °C. Once cured, the PDMS was peeled off of the mold and bonded to a flat PDMS slab via 1 min plasma treatment using the Harrick Plasma Cleaner PDC-23G (Ithaca, NY, USA). The PDMS device was then heated in an oven at 180 °C for 1 h to complete the bonding of the two polymer layers.

The basic LLC and composite samples were infused into microfluidic devices using Shenchen ISPLab01 syringe pumps (Baoding Shenchen Precision Pump Co., Ltd., Baoding city, China). All of the static and dynamic microfluidic experiments were performed at 25 °C, unless otherwise specified.

## 3. Results

### 3.1. Characterization of the Materials and Microfluidic Environment

**Figure 1:**
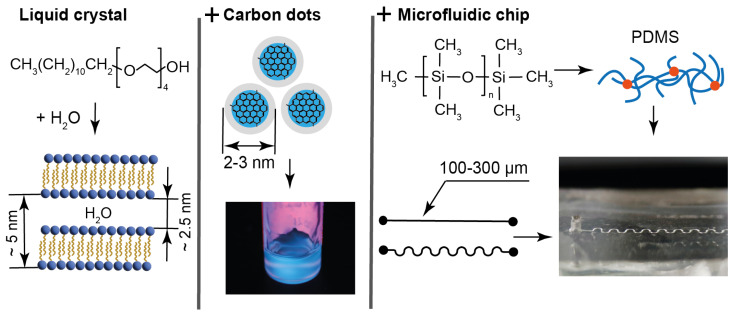
Characteristics of materials and microchips: LLC molecules and lamellar structure parameters, size characteristics, and emission properties of carbon dots in water and composites; molecular and mesh structures of cured PDMS used for fabrication of straight and serpentine microchannels of various widths.

At the first stage of this study, we summarized the characteristics of the materials and devices that were analyzed in this work, our previous works, and reported in the literature. The respective data are shown Figure 1.

Tetraethylene glycol monododecyl ether (C_12_EO_4_) was selected for producing LLCs for the following reasons: It is a water-soluble non-toxic surfactant, which forms a liquid crystalline phase in water at room temperature, which is convenient for microfluidic experiments. This LLC is a model compound, which is well studied according to the literature data [54,55,56], so its behavior can be analyzed in complex microfluidic environments without the need for additional characterization. In our previous work [57], it was demonstrated that this LLC is virtually non-luminescent. It is optically transparent and forms homogeneous luminescent composites with water-soluble carbon dots (Figure 1).

The mesophase of C_12_EO_4_ in the presence of luminescent particles was studied in previous works [58,59] using X-ray diffraction (XRD). According to XRD, C_12_EO_4_ forms a lamellar mesophase that remains stable with a sufficiently high content of luminescent additives. The lamellar phase is represented by alternating aqueous and surfactant domains, with the width of aqueous domains of 2–3 nm and about 5 nm interplanar distance (Figure 1). In combination with relatively large ethylene glycol polar groups, such aqueous domains comprise a potential hosting environment for aqueous solutions of chemicals or aqueous dispersions of small nanoparticles. These lamellae can form multilayer vesicles (lamellar onions) according to our previous studies and the literature [57,60]. Thus, the C_12_EO_4_ composite with carbon dots can be a model of biologically originated LLCs, vesicles, drug delivery systems, and cell bilayers, and possess additional luminescence capabilities.

Blue carbon dots (bCDs) were synthesized in this work by a proven hydrothermal method using citric acid and o-phenylenediamine [61]. The presence of surface functional groups in the structures of such carbon dots contributes to their good solubility in water [62,63]. The existence of such surface groups in the structures of the synthesized carbon dots was confirmed by IR spectroscopy and NMR spectroscopy data, which is provided in the Supplementary Materials.

The addition of 0.1 wt. % of the synthesized bCDs to the aqueous phase of C_12_EO_4_ was found to maintain its lamellar texture and phase transition properties and provide the composite with intensive luminescent capabilities (Figure 1 and [57]). According to transmission electron microscopy performed in the previous work [64], the synthesized carbon dots are represented by 2–3 nm nanoparticles (Figure 1). According to spectrofluorimetry experiments performed in this work for identical bCD samples, their emission peaks are 450 nm, both regarding the aqueous dispersion of bCDs and in their composite with the LLCs. Therefore, these carbon dots may be located in the hydrophilic regions of the composite, represented by aqueous domains between the C_12_EO_4_ lamellae and the boundaries formed by polar ethylene glycol groups.

Microfluidic devices were fabricated from polydimethylsiloxane (PDMS), which has been used as the global standard for microfluidics [53] and represents a biocompatible material for laboratory-scale drug delivery and organ-on-chip studies [65,66]. Its chemical and nanoscale structure is shown in Figure 1.

The contact angle of bidistilled water on the surface of the microfluidic chips used in this work was found to be 97°, which agrees with the literature [67]. The surface of PDMS is hydrophobic due to the presence of CH_3_ groups (Figure 1). After plasma activation for chip bonding, the contact angle reduced to about 57 degrees, but then returned to its original hydrophobic values of about 100° within 2–3 days.

According to the literature, PDMS forms a nanoporous mesh with 10–20 nm size pores after cross-linking [68]. Such a hydrophobic nanoporous surface can be an anchoring environment for the hydrocarbon tails of C_12_EO_4_ molecules.

The microchips were represented by straight and curved microchannels of different widths, which are the main building blocks of biomedical microfluidic devices [2,7]. According to the spectrofluorimetry experiments performed in this work, the PDMS chips demonstrated negligible luminescence. They are optically transparent and proved to have compatibility with the composite in static and dynamic experiments [57].

### 3.2. Effect of Microchannel Walls on the Intermolecular Organization of the Composite

Microfluidic channels represent a confined environment with a large surface-to-volume ratio. Evaluating the impact of their walls on the intermolecular organization of LLCs can provide valuable insights into the behavior of drug delivery systems, such as vesicles or biological media, in conditions that simulate vascular systems or exist in organ-on-chip models. At this stage of the work, we characterized the properties of the nanocomposites on microchannel walls using atomic force microscopy (AFM) and polarized optical microscopy (POM).

**Figure 2:**
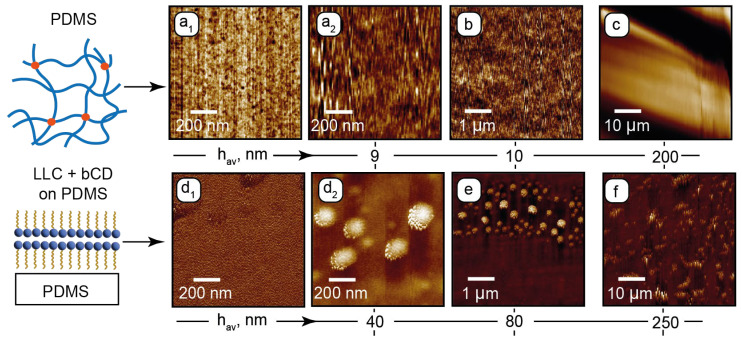
AFM surface characterization of PDMS and PDMS with the deposited composite: (**a_1_**)—sub-microscale phase image of PDMS; (**a_2_**)—sub-microscale height image of PDMS; (**b**,**c**)—microscale height images of PDMS; (**d_1_**)—sub-microscale phase image of PDMS with the deposited composite; (**d_2_**)—sub-microscale height image of PDMS with deposited composite; (**e**,**f**)—microscale height images of PDMS with the deposited composite. Height images are provided with average heights h_av_ of rough surface structures.

Figure 2 shows the AFM images for PDMS and PDMS with the deposited composite. Phase and height images of the PDMS surface in the sub-microscale range (Figure 2a_1_ and Figure 2a_2_, respectively) show nanoscale inhomogeneities in its structure. Such surface patterns agree with the literature [68,69] and can be attributed to the nanoporous structure of the cross-linked PDMS. Its surface is sufficiently flat (~10 nm roughness) in the micrometer range (Figure 2b). A periodic microscale roughness in the 50 µm range (Figure 2c) may emerge due to possible non-uniform chip-baking conditions. The resulting surface tilt evaluated from Figure 2c, however, did not exceed 1–2 degrees, thus providing relatively flat surfaces of horizontal microchannel walls.

At the next stage, a layer of the composite was deposited on the PDMS surface and dried for the AFM analysis. The residual surface structure is shown in Figure 2d_1_–f.

In the sub-microscale phase image (Figure 2d_1_), the surface shows a periodic nanoscale pattern, which can be attributed to lamellar assemblies of C_12_EO_4_. No porous structure is observed, indicating that the surfactant molecules of the composite fill the mesh pores of PDMS. The height image (Figure 2d_2_) shows 100–200 nm wide round structures. These could be the residual lamellar onions that existed in the composite and were anchored by the PDMS surface. According to the sub-microscale and microscale height images (Figure 2d_2_,e), these structures tend to anchor perpendicularly to the PDMS surface. In the 50 µm range (Figure 2f), these residual structures are tilted in the same direction, which can be the effect of shear during deposition of the sample. No nanoscale or microscale particles, which can represent bCDs or their assemblies, were revealed by AFM, indicating that carbon dots are uniformly distributed in the LLC matrix and do not tend to aggregate.

**Figure 3:**
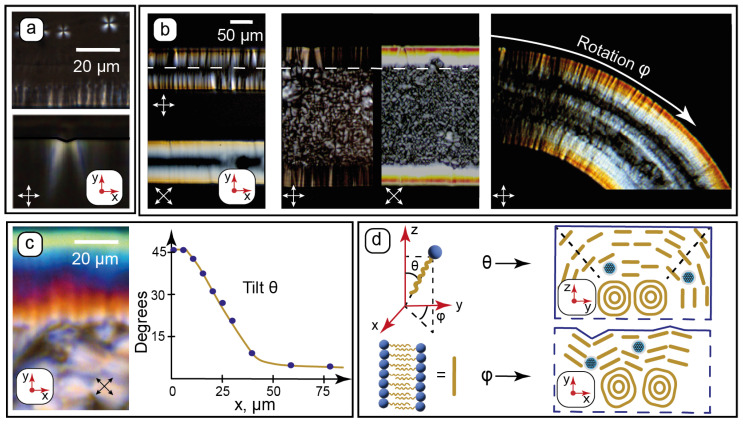
Microchannel wall effects on the molecular orientation of the composite by POM: (**a**)—characteristic texture features (lamellar onions and wall anchoring) appearing upon transition from isotropic liquid to the LLC phase at t = 60 °C; (**b**)—evaluating rotation φ of the LLC molecules in microchannels of various widths and curvatures; (**c**)—evaluating tilt θ of the LLC molecules near walls from birefringence; (**d**)—proposed alignment (not to scale) of the LLC molecules in the lamellae near channel walls from the analysis of their rotation and tilt angles. Red arrows show the coordinate axis for each section of the figure. Crossed white and black arrows show the positions of the polarizers.

Polarized optical microscopy is a powerful method which allows us not only to determine the type of liquid crystal mesophase, but also quantify the orientation of its molecules in horizontal (rotation) and vertical (tilt) planes [31,70]. In combination with ordered microfluidic confinement, such information can be valuable for characterizing the interactions of LC molecules with microchannel walls. Figure 3 summarizes the results of POM studies of the composite near microchannel walls and processing the POM data.

Wall anchoring and formation of C_12_EO_4_ lamellae in microchannels can be observed immediately after its phase transition from the isotropic liquid to the LLC phase. In the isotropic liquid phase (t > 65 °C), the composite shows no LLC texture patterns at crossed polarizers. At t < 65 °C, characteristic texture features start to appear in microchannels (Figure 3a): lamellar onions (Maltese crosses) in the middle of the microchannel and teeth-like structures at its walls. The textures developing perpendicular to the microchannel surface agree with the AFM data, which demonstrated a tendency of vertical alignment of the surface patterns (Figure 2d_2_,e).

These “teeth” patterns were found to be sensitive to the surface roughness of the vertical walls and align strongly perpendicular to the rough PDMS surface at microscale (Figure 3a). The higher roughness of vertical walls compared to the horizontal ones (Figure 2c) is related to the resolution of photolithography during the fabrication of the chips.

The intensity of light transmission through media between crossed polarizers depends on the angle between the director (averaged orientation of LLC molecules) and the polarizer [57]:

(1) $$\frac{I}{I_{0}}\sim {sin}^{2}2\varphi$$

where I and I_0_ are the transmitted and initial light intensities, respectively, and φ is the rotation angle of LLC molecules with respect to the polarizer.

Further experiments were continued at 25 °C. Surface anchoring of the LLC molecules was studied by POM for straight and curved microchannels with 100, 200, and 300 µm widths. The selected results are shown in Figure 3b. The maximum transmission was observed at crossed polarizers set at 45° to microchannel walls. According to Equation (1), this indicates a possible orientation of the LLC molecules perpendicular to microchannel walls. In the curved channel, the light transmission at its sides varies with the curvature according to Equation (1) and can be the effect of the turn of the anchored LLC molecules relative to polarizers. Such an orientation behavior can be a reasonable result of intermolecular interactions between the hydrocarbon tails of C_12_EO_4_ molecules and the hydrophobic PDMS surface. The lamellae are, therefore, assumed to align parallel to the walls.

All the images demonstrate the same characteristic anchoring patterns with ≈50 µm widths independent on the microchannel geometry (dashed white arrow in Figure 3b). They occupy nearly all of the space in the 100 µm channel, while densely packed lamellar onions are observed in central parts of wider channels. The textures near the walls, therefore, represent a gradual and continuous transition of the LLC molecular packing from the lamellar onions into the anchored lamellae at the walls. Considering that the height of all the microchannels is the same and two times higher (100 µm) than the width of the anchored patterns (≈50 µm), we can assume a symmetrical impact of vertical and horizontal walls on the surface anchoring of the LLC molecules near the wall intersections.

Comparison of the LC birefringence colors with a Michel-Levy chart [70] allows us to evaluate the vertical tilt angle of the LLC molecules from the following equation:

(2) $$\Delta n=\frac{n_{e}n_{0}}{\sqrt{{n_{e}}^{2}{sin}^{2}\theta +{n_{0}}^{2}{cos}^{2}\theta }}-n_{0}$$

where Δn is birefringence, n_e_ and n_0_ are anisotropic refractive indices, and θ is the tilt angle.

The symmetrical impact of microchannel walls on molecular anchoring of the LLCs can be used to solve Equation (2). We can assume that the tilt angle is θ ≈ 45° near the intersection of the walls (edge of the channel). The birefringence Δn in this region was evaluated by comparing the respective channel edge color in Figure 3c with the Michel-Levy chart, which was reproduced in Matlab according to [52]. The average refractive index of the composite measured in this work by refractometry was found to be n = 1.4030. Taking into account the birefringence relationship n ≈ (n_e_ + 2n_0_)/3 [71], we can solve Equation (2) and evaluate all of the variables.

Equation (2) was solved in Matlab. Then, tilt angles were calculated by comparing birefringence colors within 100 µm from the microchannel vertical wall (Figure 3c) with the Michel-Levy chart. The plot in Figure 3c demonstrates the resulting approximate average tilt angles. The tilt was found to decrease smoothly from the assumed 45° at the edge to constant 7–8° in the central part of the microchannel. This residual average tilt can be attributed to the competition between intermolecular forces governing the curved multilayer alignment of the LLC molecules in lamellar onions and the anchoring of the LLC molecules by the PDMS surface.

Finally, Figure 3d summarizes the proposed orientation of the LLC molecules arranged into lamellae with respect to the microchannel walls. C_12_EO_4_ molecules are anchored by the hydrophobic PDMS surface, and the lamellae are assumed to align parallel to the microchannel walls. Thus, microfluidic anchoring of the nanocomposite was found to be governed by three main factors: the molecular structure of PDMS (hydrophobic surface), microscale channel roughness, and the symmetrical anchoring impact of intersecting vertical and horizontal walls.

### 3.3. Effect of Flow Dynamics on the Intermolecular Organization of the Composite

**Figure 4:**
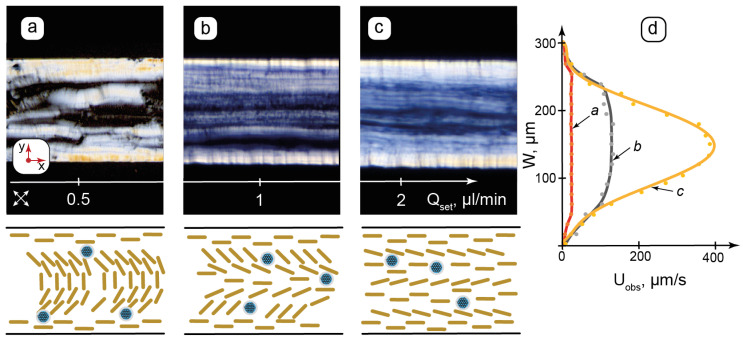
Flow-induced transition from lamellar onions of the composite to the axial lamellar structure in microchannels by POM and proposed (not to scale) schematic orientation of the lamellae: (**a**)—realignment of lamellar onions with respect to shear; (**b**)—decomposition of lamellar onions; (**c**)—transition to a planar lamellar texture; (**d**)—respective flow velocity profiles for (a, b, c). Q_set_—flow rate set by syringe pump; U_obs_—observed flow velocity. Red arrows show the coordinate axis for each section of the figure. Crossed arrows show the positions of the polarizers.

In addition to the specific impact of microchannel walls, precisely controlled flow dynamics is the second key factor that primarily distinguishes microfluidic confinement from macroscopic conditions. Analyzing the flow of organized media in microchannels can provide valuable information about their shear-induced behavior and possible structural transformations. The flows of the composite in microchannels of various geometries were studied by POM. The selected results are shown in Figure 4.

Microfluidic syringe pumps allow us to set the flow rate, which is proportional to the pressure applied to the microchannel inlet by the syringe through a connecting tube. The composite was studied at the set flow rates up to 5 µL/min in both linear and curved channels. The selected results for the linear 300 µm channels are shown in Figure 4a–c. To determine flow velocity profiles in the microchannel, sets of images were obtained by capturing frames at specific time intervals. Changes in the positions of the texture fragments between the frames were analyzed to plot the velocity distribution for the set flow rates (Figure 4d).

Figure 4a shows a texture with a distinct side wall anchoring. A relatively disordered texture with short bright sections and spots in the central part of the channel can represent the shear-induced rearrangement of the lamellae, which were initially vertically anchored to the horizontal walls. Such sheared textures can be compared with large-scale AFM images (Figure 2f), where the residual structures were tilted in the same direction due to shear during deposition.

While the flow velocity of the “a” sample (Figure 4d) decreases to zero at the walls, it has nearly the same value elsewhere (≈30 µm/s) and is far below the expected average flow velocity of ≈300 µm/s, which can be calculated by dividing the set flow rate to the microchannel’s cross-section. In such conditions, therefore, the applied pressure and the resulting shear are sufficient to deform the lamellar ions, but still too weak to overcome intermolecular interactions and break the packing of LLC molecules in these interconnected multilayer vesicular structures.

Figure 4b demonstrates the texture of alternating dark and bright stripes along the flow direction and the remaining light transition maximum at vertical walls, indicating strong wall anchoring. According to Equation (1), rotation angles of the LLC molecules vary consecutively in such alternating stripes. A slightly curved pattern appears in the flow velocity profile (Figure 4d), indicating the differences appearing in flow velocities of contacting lamellar layers. Figure 4b, therefore, represents the transition between the decomposing lamellar onions and the appearing flow-aligned lamellar threads in the microchannel.

In Figure 4c, the central texture is aligned along the flow, although the light transmittance is not completely uniform. At this flow rate, the velocity distribution profile is close to parabolic in the center, while it still decreases abruptly at the walls. Figure 4c, therefore, marks the end of the transition from the lamellar onions to the planar lamellar structure with a relatively non-hindered motion of the lamellar threads. The wall anchoring of LLC molecules to the PDMS surface, however, still dominates the shear impact. With further increases in the flow rate up to Q_set_ = 5 µL/min, the real flow velocity in the microchannel center approaches its expected value set by the syringe pump flow rate, corresponding to the parabolic flow profile. The wall anchoring effects virtually disappear.

Similar flow behavior of the composite was observed in microchannels of different widths (100–300 µm) and geometries (straight and serpentine). The flow behavior of the pure LLC phase without carbon dots was found to be nearly the same.

The flow-induced transformations of the composite were found to be reversible. Applying a heating–cooling cycle with the phase transition to isotropic liquid and further cooling down to room temperature allows us to return to non-sheared textures, as shown in Figure 3b, and repeat the flow-induced transformation of lamellar onions and wall-anchored structures into axial lamellar threads.

Thus, flow dynamics in microchannels allows us to perform controlled transitions between various types of supramolecular organization of the nanocomposite. In combination with heating–cooling cycles, such a controlled transformation can be performed reversibly and repeatedly.

### 3.4. Luminescent Properties of the Composite in Microfluidic Environment

**Figure 5:**
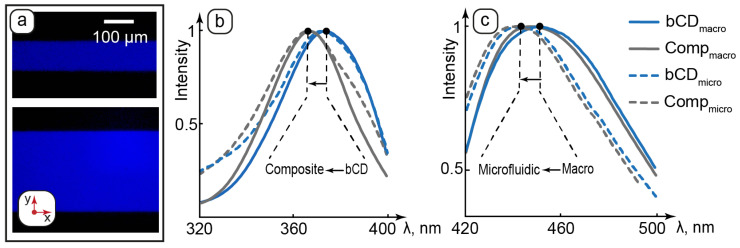
Luminescent properties of bCDs and the composite: (**a**)—fluorescence microscopy images of the composite in microchannels of various geometries; (**b**)—normalized excitation spectra of carbon dots and the composite in cuvette (macro) and microchannels (micro); (**c**)—normalized emission spectra of carbon dots and the composite in cuvette (macro) and microchannels (micro).

Hybrid anisotropic and luminescent organized media are potent materials for biomedical applications. Analysis of their luminescent properties in different conditions can contribute to characterizing their structural changes and respective intermolecular interactions. At this stage of the work, we studied the luminescent properties of bCDs and composites in macroscopic and luminescent conditions. Fluorescence microscopy and spectroscopy data are combined in Figure 5.

The composite generated stable, intensive, and homogeneous blue color emission in microchannels of all the studied geometries at all the studied set flowrates (Figure 5a shows the selected images). These results agree with AFM, which demonstrated no microscale aggregation of bCDs in the composite. The emission is also uniform across the microchannels. Therefore, no considerable wall effects in the luminescence of bCDs were detected, and their distribution in the water pool of the LLC matrix of the composite can be more preferable than interactions with hydrophobic PDMS surfaces due to the hydrophilic nature of the studied carbon dots.

Comparison of the excitation spectra of bCDs and the composite revealed a ~10 nm shift in the absorbance peaks of both the macroscopic and microfluidic samples to the short-wave area (Figure 5b). The LLCs demonstrated negligible absorbance and emission as compared to bCDs. Growth in the short-wave absorption of bCDs in the composite can be caused by changes in its surrounding environment of carbon dots upon their transfer to the hosting matrix of the composite. The presence of hydrophilic groups on the surface of bCDs assumes their preferred interactions with polar molecular groups such as ethylene glycol head groups of surfactant molecules, which separate aqueous domains from nonpolar hydrocarbon layers in the lamellar phase. According to the literature, the shift to the short-wave area observed for carbon dots can be attributed to intermolecular interactions such as hydrogen bonding [72,73]. Carbon dots are, therefore, considered to be located in the aqueous domains of the lamellae and participate in intermolecular interactions with the surrounding environment, such as possible hydrogen bonding with polar groups of LLC molecules.

Both bCDs and the composite showed a slight shift in the emission peak to the short-wave area in microchannels (Figure 5c). This effect can be attributed to a slight scattering of emission light by the microchip.

Shear-induced textures in the dynamic composite, shown in Figure 4, were found to remain virtually the same within about 30 min after stopping the flow due to the relatively high viscosity of the composite. It also allowed us to study luminescence characteristics of the samples realigned using flow dynamics by placing the chips with the sheared composite into the spectrofluorimeter compartment.

An important parameter of luminescent media is the possible anisotropy of their light emission, which can be useful in biomedical applications for characterizing molecular alignment [74,75]. The luminescence anisotropy of bCDs and the composite was evaluated using polarized spectrofluorimetry data according to [75].

The excitation and emission peaks and the respective luminescence anisotropy values of both static and dynamic samples are summarized in Table 1.

According to Table 1, the same differences in the excitation and emission peaks observed in the static composite (Figure 5) also remained in the dynamic samples. This indicates that the respective impact of shear did not result in the significant rearrangement of surrounding molecular environment around bCDs, which could affect their luminescence behavior.

The luminescence anisotropy of macroscopic bCDs, both in water and the composite (samples 1 and 2), was found to be negligible compared to the theoretical maximum of 0.4 [74]. The slightly higher anisotropy, demonstrated by the microfluidic aqueous dispersion of carbon dots (sample 3), could be related to a possible impact of the microchip’s material and geometry on the experimental results.

The relatively high anisotropy of the static microfluidic composite (sample 4) can be attributed to anchoring LLC molecules by the walls and the resulting development of ordered anisotropic environment throughout a microchannel. The slight drop of anisotropy, as demonstrated by the slowly sheared sample 5, indicates the effect of rearrangement and decomposition of the lamellar onions and the decrease in anisotropic ordering. Finally, sample 6 demonstrated the highest anisotropy of luminescence, which could be the result of the ordered shear-induced axial orientation of the lamellae.

The measured relative quantum yield of luminescence was found to be 25.5% for the aqueous dispersion of bCD. A limitation of this study was the fact that it was not possible to calculate the quantum yield for the composite due to a more intensive light scattering and difficulties in obtaining an adequate absorption spectrum.

An important question that should be explained in more detail is the origin of the anisotropic luminance of the composite. Certain alignment of carbon dots in the hosting environment, which can result in anisotropic luminance, may be assumed according to their interactions within the liquid crystalline matrix. Such interactions can be related to the shift in spectrofluorimetry peaks, shown in Figure 5. In the spectrofluorimetry experiments, however, the macroscopic sample of the composite demonstrated negligible anisotropy of luminescence, according to Table 1. Therefore, although carbon dots can interact with the host, this interaction does not necessarily result in anisotropic luminance.

In the microfluidic samples, ordered orientation of the composite by walls and flow created an environment with carbon dots embedded in the ordered anisotropic LLC matrix and provided increased luminescence anisotropy. The resulting alignment of carbon dots interacting with the molecules of the more ordered LLC matrix may contribute to the luminescence anisotropy. The primary contributor to the luminescence anisotropy, however, is considered to be the microchannel-governed alignment of the hosting LLC matrix.

Thus, dynamic and wall effects of the microfluidic confinement allowed us to control the luminescence anisotropy of the studied C_12_EO_4_—bCD composite.

### 3.5. Application of Machine Learning Tools to Analyzing the Behavior of Composites in Microchannels

**Figure 6:**
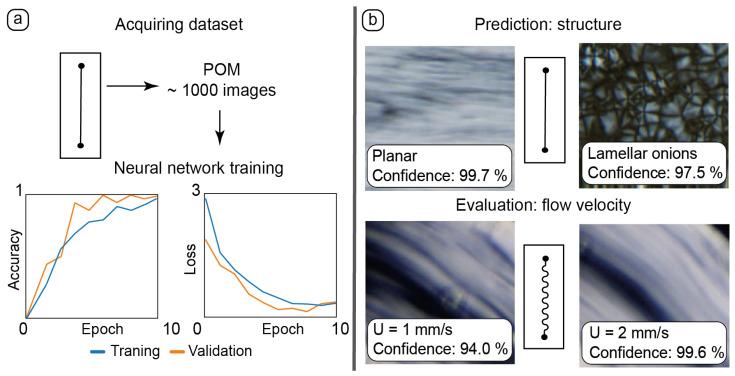
Application of AI tools to processing experimental data: (**a**)—training the neural network with the dataset of 1000 POM images of static and dynamic microfluidic experiments in the straight chip; (**b**)—using these training results for new experimental data: predicting LLC alignment and evaluating its flow velocity in chips of various geometries.

Combinations of artificial intelligence tools and microfluidic devices has offered new opportunities for the optimization of microfluidic processes and control of organized media properties in the last 2–3 years [76,77]. Experimental datasets obtained in an ordered microchannel environment are convenient for training neural networks and making conclusions, which are too labor-intensive or time-consuming without using AI. The last part of this work focuses on using microfluidic POM datasets for training a neural network for recognizing the molecular ordering of the LLCs and the flow velocity of the composite in curved microchannels. The results are summarized in Figure 6.

Experimental datasets were collected by capturing static and dynamic POM images in various parts of microchannels (Figure 6a). A dataset of ≈1000 images was sufficient to provide adequate results of training accuracy and validation.

After training, the neural network was tested with new static images representing different textures of LLCs (Figure 6b, top). The resulting accurate recognition allowed for the neural network to detect various LLC structures in microchannels such as flow-aligned lamellar threads in planar samples and lamellar onions.

Manual evaluation of the flow velocity in straight channels turned out to be a time-consuming process requiring a thorough analysis of microscale LLC textures. The geometry of curved channels made this process even more complicated.

The results of manual flow velocity measurements in straight channels were transformed into a dataset, with blocks of images corresponding to the set flow rate and respective flow velocity. The results of neural network training were applied to images of dynamic microflows in curved channels (Figure 6b, bottom). The neural network performed an adequate evaluation of the flow velocities in curved channels based on delicate changes in the respective LLC textures.

To summarize, a machine learning approach was successfully applied to the recognition of LLC supramolecular organization in microchannels and the evaluation of the flow velocity of the composite in complex curved-channel geometries.

## 4. Discussion

The results of this work demonstrate the predominant impact of surface and dynamic effects on the intermolecular organization of the luminescent composite of blue carbon dots and lyotropic liquid crystals. Nanoscale and microscale wall effects created a complex and symmetrical three-dimensional organization of the nanocomposite in confined microchannel space.

Molecular anchoring at the microchannel surface resulted in the formation of ordered microscale structures that did not exist in non-microfluidic conditions. Anchored lamellar onions represent intermolecular structures of a hybrid geometry, with a gradual and continuous transition from close-to-spherical packing of lamellar layers into lamellar layers, which tend to stack parallel to the walls. On the other hand, microflow dynamics offers a controlled and reversible transition from the closed-loop molecular packing of lamellar onions to the open texture of axial lamellar threads.

Surface and dynamic ordering of the composite in microchannels allowed us to create anisotropic luminescent media from the substances, which demonstrated the negligible anisotropy of luminescence in non-microfluidic conditions. Intensive luminescence of carbon dots made it possible to combine microfluidic chips with standard macroscopic techniques such as spectrofluorimetry.

As compared to macroscopic data shown in our previous work [55], microfluidic experimental results such as POM images are more specific and diverse, which makes them more convenient for training neural networks. The AI approach proved to be fruitful for both recognizing the nanoscale structure of the composite from its images in microchannels and evaluating parameters such as flow velocity, which are too complicated for characterization without AI tools.

The resulting advantages of microfluidic devices with integrated luminescent nanocomposites highlight several trends for their potential application. The microchips with an integrated LLC matrix and bCDs can be used to create in-channel media with quantitatively controlled polarized luminescence for photonic applications such as the analysis of luminescent markers in lab-on-chip devices. Such nanocomposites can represent models of biological and drug delivery systems (vesicles, cell membranes, surfactant bilayers). Analyzing their in-channel behavior can be helpful for characterizing the properties of such systems in conditions that are close to biological vascular systems or that exist in organ-on-chip prototypes. The compatibility of microfluidic chips using the machine learning approach is promising for developing AI-powered chips for real-time monitoring of the luminescent nanocomposite transformations in organized microfluidic confinement.

Future research plans will focus on broadening the range of LLC substances: characterizing nanocomposites of carbon dots with LLCs that form hexagonal or nematic mesophase in microchannels and incorporating carbon dots with other spectral characteristics. The AI tools are intended to be adapted to real-time analyses of LLC behavior in microchips.

## 5. Conclusions

A combination of surface effects, flow dynamics, and phase transitions in microfluidic channels allowed us to create a set of ordered intermolecular structures in the composite of the studied lyotropic liquid crystal and blue carbon dots, which do not exist in non-microfluidic conditions. Surface anchoring of this hybrid material eliminates nanoporous roughness of PDMS and creates symmetrical 3D textures with a continuous transformation of multilayer vesicles into stacks of lamellae aligned parallel to microscale surface patterns of the walls. Flow dynamics allows us to reversibly transform such structures using a step-by-step decomposition of the lamellar onions into axial lamellar threads. Surface and dynamic effects provide microfluidic composites of lyotropic liquid crystals and carbon dots with controlled luminescence anisotropy capabilities. The neural network learning approach demonstrated its efficiency in processing large experimental datasets of approximately 1000 microscopy images for predicting the nanoscale organization of the studied composite and evaluating its flow velocity in curved channels with complex geometry.

The discussed surface and dynamic effects of the studied composite highlight its potential applications in anisotropic luminescent media, materials for microfluidic photonics, and models of biological and drug delivery systems in confined networks of organ-on-chip channels.

## Figures and Tables

**Table 1:** Luminescent properties of carbon dots and the composite in macroscopic and microfluidic conditions.

Sample	Conditions	System	Q_set_, µL/min *	POM Image	Excitation Peak, nm	Emission Peak, nm	Average Anisotropy
1	Cuvette	bCD + H_2_O	-	-	365	449	0.02
2	Cuvette	Composite	-	-	374	450	0.02
3	Chip	bCD + H_2_O	-	-	365	442	0.08
4	Chip	Composite	0	Figure 3b	374	440	0.16
5	Chip	Composite	0.5	Figure 4b	374	441	0.12
6	Chip	Composite	2	Figure 4c	373	443	0.25

* Luminescence spectra were acquired after stopping the flow.

## Data Availability

The original contributions presented in this study are included in the article/Supplementary Material. Further inquiries can be directed to the corresponding authors.

## Supplementary Materials

The following supporting information can be downloaded at: https://www.mdpi.com/article/10.3390/nano15211682/s1.

## Abbreviations

The following abbreviations are used in this manuscript:

LC	Liquid crystal
LLC	Lyotropic liquid crystal
bCDs	Blue carbon dots
POM	Polarized optical microscopy
XRD	X-ray diffraction
C_12_EO_4_	Tetraethylene glycol monododecyl ether
PDMS	Polydimethylsiloxane
AI	Artificial intelligence
