# Supplementary material for: Intermolecular Organization of a Lyotropic Liquid Crystal and Carbon Dot Composite in Microfluidic Channels: Surface and Dynamic Effects
Source: Nanomaterials (Basel). 2025 Nov 6;15(21):1682. doi: 10.3390/nano15211682 (PMC12610427; doi:10.3390/nano15211682)
Supplement: Supplementary file 1 [file nanomaterials-15-01682-s001.zip › nanomaterials-3951791-supplementary.pdf]

Characterization of carbon dots

Characterization of surface groups of carbon dots was performed by IR spectroscopy (Fig. S1), and ^{1}H and ^{13}C NMR spectroscopy (Fig. S2). The spectra demonstrate the presence of various surface groups in addition to the aromatic framework of benzene rings.

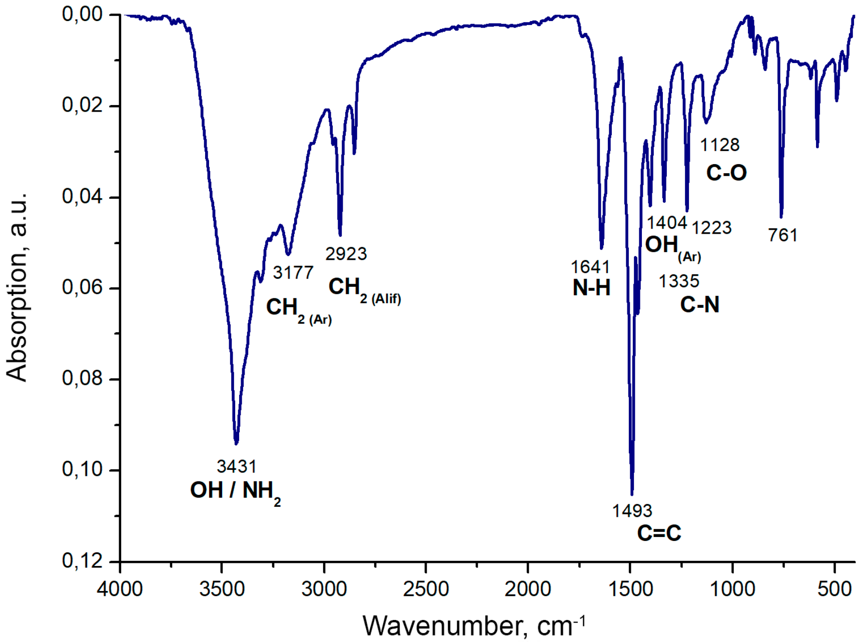

Figure S1. IR spectra of carbon dots.

The IR spectra contain bands at 3431, 1641 and 1335 cm^{-1}, which correspond to vibrations of NH_2, N-H and C-N bond, respectively. Therefore, the synthesized carbon dots include nitrogen-containing surface groups. The bands at 3177 and 1493 cm^{-1} indicate vibrations of aromatic carbon bonds. This confirms formation of graphene-like structure of carbon dots. The band at 761 cm^{-1} can be attributed to vibrations of mono and 1,2- substituted aromatic compounds.

NMR data (Fig. S2) also confirm formation of aromatic structures functionalized by surface groups.

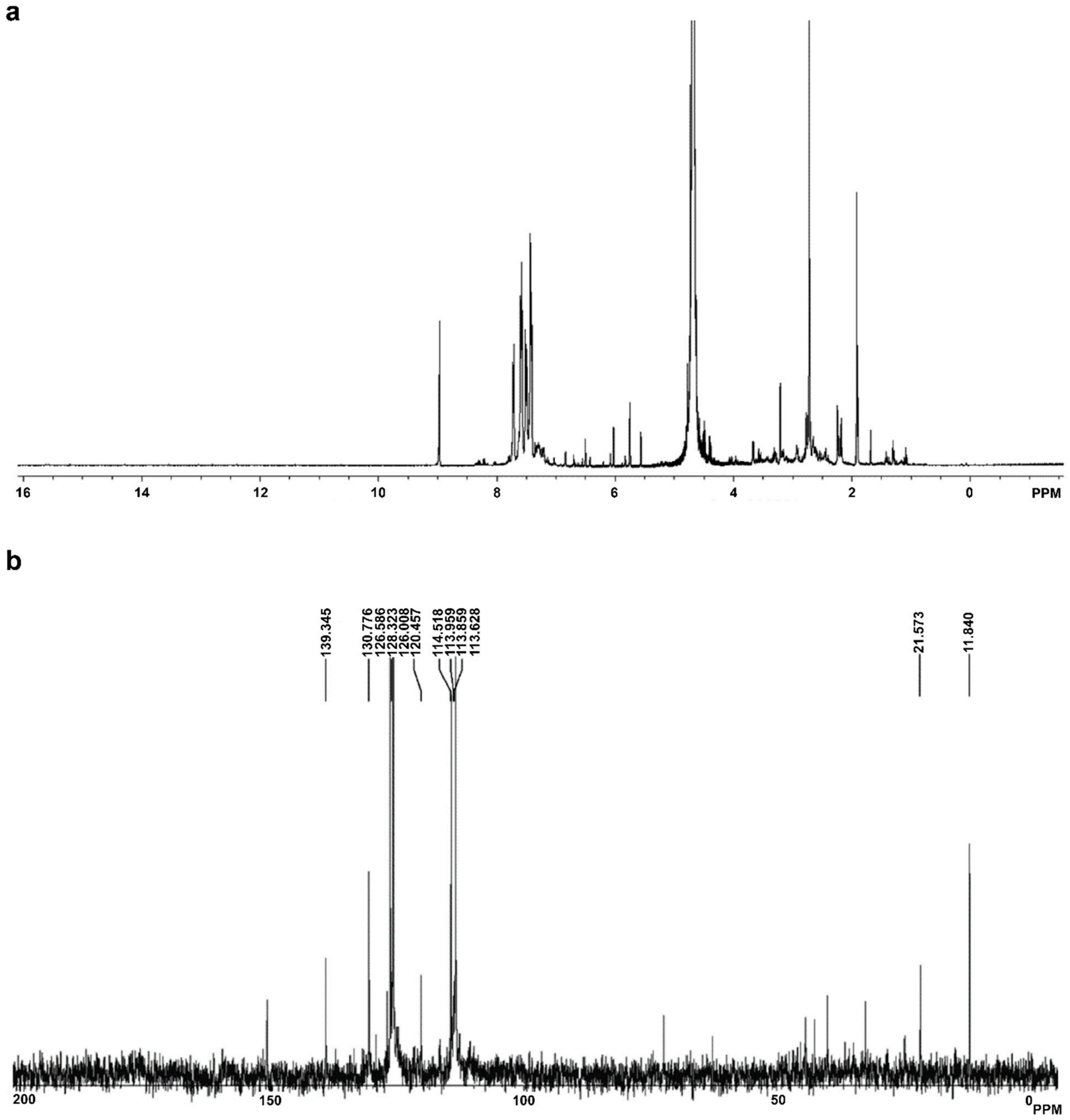

Figure S2. ^{1}H (a) and ^{13}C (b) NMR spectra of the synthesized carbon dots.

The ^{1}H NMR spectrum contains chemical shift at 1,908 ppm, which can be attributed to R-CH_3 hydrogen atoms, while the 2,727 ppm shift can be attributed to Ar-CH_2-R hydrogen atoms. The shifts at 4,464 and 8,968 ppm can be attributed to surface and aromatic amino groups. Ether groups are represented by the shift at 8,968 ppm.

In the ^{13}C (b) NMR spectra, the bands within 120-130 ppm correspond to vibrations of benzene rings. The bands at 10-50 ppm can be attributed to methyl groups, while the 150 ppm peak may characterize carbonyl surface groups.

IR spectrum was obtained by ALPHA-T S/N 102706 (Bruker) Fourier spectrometer with the spectral range of 4000-370 cm^{-1} and resolution of 4 cm^{-1} in KBr.

The ^{1}H and ^{13}C NMR spectra were recorded by Bruker Avance-400 [400.0 (^{1}H), 100.6 (^{13}C) MHz], in D_2O at 25°C with respect to the signals of residual protons or deuterium nuclei of the solvent (^{1}H and ^{13}C).
